# Supplementary material for: Severity of Hepatocyte Damage and Prognosis in Cirrhotic Patients Correlate with Hepatocyte Magnesium Depletion
Source: Nutrients. 2023 Jun 3;15(11):2626. doi: 10.3390/nu15112626 (PMC10255194; doi:10.3390/nu15112626)
Supplement: Supplementary file 1 [file nutrients-15-02626-s001.zip › Supplementary table S1.pdf]

***Supplementary table S1.*** *Demographic and clinical characteristics of the cirrhotic patients of the study populations A and B, subgrouped according to the respective median of MELDNa*

| | Cirrhotics of the study population A with low MELDNa | Cirrhotics of the study population A with high MELDNa | P value | Cirrhotics of the study population B with low MELDNa | Cirrhotics of the study population B with high MELDNa | P value |
|---|---|---|---|---|---|---|
| **Age, median (IQR)** | 55.5<br>(48.2-62) | 51<br>(45-56) | 0.200 | 55.7<br>(52.3-63.8) | 57.7<br>(52-61.9) | 0.861 |
| **Sex, male, n (%)** | 10 (83.3) | 12 (80) | 1 | 14 (93.3) | 13 (81.3) | 0.600 |
| **BMI (Kg/m²), median (IQR)** | 23.2<br>(22.6-30.6) | 24.4<br>(23-26.9) | 0.755 | 25.1<br>(24.4-29) | 26.7<br>(23.5-28.9) | 0.572 |
| **Cirrhosis aetiology, n (%):**<br>**MAFLD**<br>**Alcohol**<br>**Viral** | <br>7 (58.3)<br>5 (41.6)<br>9 (75) | <br>9 (60)<br>5 (33.3)<br>10 (66.6) | <br>1<br>0.706<br>0.696 | <br>7 (46.6)<br>8 (53.3)<br>6 (40) | <br>10 (62.5)<br>8 (50)<br>6 (37.5) | <br>0.376<br>0.853<br>0.886 |
| **HCC, n (%)** | 11 (91.6) | 3 (20) | <0.001 | 15 (100) | 2 (12.5) | <0.001 |
| **Diuretic treatment, n (%):**<br>**No**<br>**K sparing diuretics only**<br>**Loop diuretics plus K sparing diuretics.** | <br>6 (50)<br>4 (33.3)<br>2 (6.7) | <br>2 (13.3)<br>12 (80.0)<br>1 (6.7) | 0.031 | <br>8 (53.3)<br>4 (26.7)<br>3 (20.0) | <br>3 (35.5)<br>4 (12.9)<br>16 (51.6) | 0.001 |
| **MELDNa, median (IQR)** | 13.5 (11-15) | 20 (19-27.8) | <0.001 | 12 (8-14) | 22 (22.3-28.8) | <0.001 |
| **Serum AST (IU/L), median (IQR)** | 41(29-70) | 90 (47-112) | 0.007 | 35 (29-72) | 84.5 (56.8-139.5) | 0.005 |
| **Serum ALT (IU/L), median, (IQR)** | 37.5 (30.5-57) | 51 (25-76) | 0.456 | 36 (23-58) | 53.5 (33.3-82) | 0.119 |
| **Serum Mg, mg/dL, median (IQR)** | NAV | NAV | - | 1.9 (1.7-2.1) | 1.9 (1.6-1.94) | 0.318 |
| **Tissue Mg, (µg/g), median, (IQR) *** | 125.8 (116.8-141.1) | 113.2 (101.7-123.2) | 0.005 | NAV | NAV | - |
| **Percentage of weak positive hepatocytes to TRPM7 stain, median (IQR)** | NAV | NAV | - | 46.4 (26.4-54.2) | 50.6 (36.3-57.8) | 0.247 |
| **Percentage of intense positive hepatocytes to TRPM7 stain, median, (IQR)** | NAV | NAV | - | 0.8 (0-1.8) | 9 (3.8-17.3) | <0.001 |
| **Percentage of total positive hepatocytes to** | NAV | NAV | - | 47.2 (24.8-55) | 55.9 (51.5-75.7) | 0.014 |

| | | | | | | | | |
|---|---|---|---|---|---|---|---|---|
| **TRPM7 stain, median (IQR)** | | | | | | | | |

* Measured by atomic absorption. All data refer to the time of liver transplant, unless otherwise specified.
Abbreviations: AST aspartate amino transferase; ALT alanine amino transferase; BMI: body mass index; HCC, hepatocellular carcinoma; IQR, interquartile range; MAFLD, metabolic associated fatty liver disease;  MELDNa, Model for End stage Liver Disease Sodium; K, potassium; MELD, Mg, magnesium; NAV, not available; TRPM7, Transient Receptor Potential Cation Channel Subfamily M Member 7.
