# Supplementary material for: Severity of Hepatocyte Damage and Prognosis in Cirrhotic Patients Correlate with Hepatocyte Magnesium Depletion
Source: Nutrients. 2023 Jun 3;15(11):2626. doi: 10.3390/nu15112626 (PMC10255194; doi:10.3390/nu15112626)
Supplement: Supplementary file 1 [file nutrients-15-02626-s001.zip › Supplementary table S2 .pdf]

Supplementary table S2. Demographic and clinical characteristics of the cirrhotic patients of the study populations A and B, subgrouped according to the respective median value of serum AST activity.

	Cirrhotics of the study population A with low serum AST	Cirrhotics of the study population A with high serum AST	P value	Cirrhotics of the study population B with low serum AST	Cirrhotics of the study population B with high serum AST	P value
Age, median (IQR)	55.0 (48.0-61.0)	50.5 (47.3-57.8)	0.519	60.0 (55.6-63.8)	54.9 (50.1-60.2)	0.054
Sex, male, n (%)	11 (84.6)	11 (78.6)	1	13 (86.7)	14 (87.5)	1
BMI (Kg/m^2), median (IQR)	23.1 (22.6-29.6)	24.8 (23.3-27.4)	0.488	25.9 (24.4-29.8)	25.5 (23.5-27.6)	0.572
Cirrhosis aetiology, n (%):						
MAFLD	7 (53.8)	9 (64.3)	0.704	9 (60)	8 (50)	0.576
Alcohol	6 (46.2)	4 (28.6)	0.440	6 (40)	10 (62.5)	0.210
Viral	8 (61.5)	11 (78.6)	0.420	4 (26.7)	8 (50.0)	0.273
HCC, n (%)	9 (69.2)	5 (35.7)	0.128	11 (73.3)	6 (37.5)	0.073
Diuretic treatment, n (%):			0.637			0.102
No	5 (38.5)	3 (21.4)		4 (26.7)	7 (43.8)	
K sparing diuretics only	7 (53.8)	9 (64.3)		4 (26.7)	0 (0)	
Loop diuretics plus K sparing diuretics	1 (7.7)	2 (14.3)		7 (46.7)	9 (56.3)	
MELDNa, median (IQR)	14.0 (11.2-22.0)	19.0 (17.8-22.3)	0.054	14.0 (10.0-18.0)	24.0 (14.8-27.8)	**0.006**
Serum AST (IU/L), median (IQR)	40.0 (30.0-44.0)	98.0 (76.8-115.3)	**<0.001**	33.0 (28.0-46.0)	97.0 (72.8-139.8)	**<0.001**
Serum ALT (IU/L), median, (IQR)	30.0 (24.0-37.5)	59.5 (50.0-100.0)	**<0.001**	25.0 (20.0-37.0)	61.0 (44.3-84.8)	**<0.001**
Serum Mg, mg/dL, median (IQR)	NAV	NAV	-	1.90 (1.70-2.11)	1.90 (1.61-1.94)	0.358
Tissue Mg, (µg/g), median, (IQR) *	118.7 (114.3-140.6)	114.1 (106.9-125.8)	0.220	NAV	NAV	-

Percentage of weak positive hepatocytes to TRPM7 stain, median (IQR)		NAV	NAV	-		43.2 (24.6-52.4)	50.6 (36.3-57.8)	0.118
Percentage of intense positive hepatocytes to TRPM7 stain, median, (IQR)		NAV	NAV	-		0.8 (0.2-4.0)	7.0 (1.5-12.5)	**0.041**
Percentage of total positive hepatocytes to TRPM7 stain, median (IQR)		NAV	NAV	-		47.2 (24.8-56.2)	55.0 (50.9-74.7)	0.060

* Measured by atomic absorption. All data refer to the time of liver transplant, unless otherwise specified

Abbreviation: AST aspartate amino transferase; ALT alanine amino transferase; BMI: body mass index; HCC, hepatocellular carcinoma; IQR, interquartile range; MAFLD, metabolic associated fatty liver disease; MELDNa, Model for End stage Liver disease with added serum sodium concentration parameter; Mg, magnesium. NAV, not available
