# Supplementary material for: Severity of Hepatocyte Damage and Prognosis in Cirrhotic Patients Correlate with Hepatocyte Magnesium Depletion
Source: Nutrients. 2023 Jun 3;15(11):2626. doi: 10.3390/nu15112626 (PMC10255194; doi:10.3390/nu15112626)
Supplement: Supplementary file 1 [file nutrients-15-02626-s001.zip › Supplementary table S3.pdf]

Supplementary table S3. Demographic and clinical characteristics of the cirrhotic patients of the study populations A and B, subgrouped according to the respective median value of Δ-MELDNa during the waitlist time

	Cirrhotics of the study population A with low Δ-MELDNa	Cirrhotics of the study population A with high Δ-MELDNa	P value	Cirrhotics of the study population B with low Δ-MELDNa	Cirrhotics of the study population B with high Δ-MELDNa	P value
**Age, median (IQR)**	51.0 (45.0-59.0)	54.0 (49.0-60.8)	0.239	56.5 (52.3-60.3)	59.1 (52.1-63.7)	
**Male sex, n (%)**	10 (76.9)	12 (85.7)	0.648	12 (80.0)	15 (93.8)	
**BMI (Kg/m^2), median (IQR)**	24.1 (22.6-28.4)	24.3 (22.9-26.7)	0.943	25.9 (24.0-29.7)	25.7 (24.5-28.7)	
**Cirrhosis aetiology, n (%):**						
**MAFLD**	7 (53.8)	9 (64.3)	0.704	9 (60.0)	8 (50.0)	0.576
**Alcohol**	5 (38.5)	5 (35.7)	1	10 (66.7)	6 (37.5)	0.156
**Viral**	9 (69.2)	10 (71.4)	1	4 (26.7)	8 (50.0)	0.273
**HCC, n (%)**	8 (61.5)	6 (42.9)	0.449	9 (60.0)	8 (50.0)	0.576
**Diuretic treatment, n (%):**			0.331			0.621
**No**	4 (30.8)	4 (28.6)		5 (33.3)	6 (37.5)	
**K sparing diuretics only**	0 (0)	3 (21.4)		1 (6.7)	3 (18.8)	
**Loop diuretics plus K sparing diuretics**	9 (69.2)	7 (50.0)		9 60.0)	7 (43.8)	
**MELDNa at transplant, median (IQR)**	16.0 (12.2-23.0)	19.0 (14.8-21.8)	0.402	14.0 (10.0-23.0)	22.0 (14.0-28.8)	**<0.001**
**MELDNa at listing, median (IQR)**	15.9 (12.2-25.7)	14.6 (11.4-17.8)	0.280	15.9 (12.8-22.5)	17.6 (9.8-28.8)	0.830
**Δ-MELDNa, median (IQR)**	0.0 (-1.31 – 0.0)	1.09 (0.24-3.51)	0.059	-0.31 (-1.84-0.08)	3.23 (1.34-10.83)	**<0.001**
**Serum AST (IU/L), median (IQR)**	41.0 (35.5-70.0)	88.0 (58.5-115.3)	**0.009**	43.0 (28.0-59.0)	93.0 (61.0-139.5)	**0.001**
**Serum ALT (IU/L), median, (IQR)**	32.0 (24.5-59.0)	51.0 (43.5-95.3)	0.089	29.0 (20.0-48.0)	59.5 (36.3-84.8)	**0.002**
**Serum Mg, mg/dL, median (IQR)**	NAV	NAV	-	1.90 (1.70-2.11)	1.75 (1.63-1.94)	0.188
**Tissue Mg, (µg/g), median, (IQR) ***	118.7 (113.3-140.6)	114.7 (102.8-125.8)	0.280	NAV	NAV	

**Percentage of weak positive hepatocytes to TRPM7 stain, median (IQR)**	NAV	NAV	-	42.8 (24.6-56.2)	50.2 (40.7-56.3)	0.299
**Percentage of intense positive hepatocytes to TRPM7 stain, median, (IQR)**	NAV	NAV	-	0.6 (0.0-4.0)	7.0 (1.8-17.3)	**0.001**
**Percentage of total positive hepatocytes to TRPM7 stain, median (IQR)**	NAV	NAV	-	46.8 (24.8-56.2)	55.4 (50.9-73.6)	0.045

*** Measured by atomic absorption. All data refer to the time of liver transplant, unless otherwise specified**

Abbreviation: AST aspartate amino transferase; ALT alanine amino transferase; BMI: body mass index; HCC, hepatocellular carcinoma; IQR, interquartile range; K, potassium; MAFLD, metabolic associated fatty liver disease; MELD, model of end stage liver disease; MELDNa, MELD with added a serum sodium concentration parameter; Mg, magnesium; NAV, not available; Δ, delta.
