# Supplementary figures and images for: Severity of Hepatocyte Damage and Prognosis in Cirrhotic Patients Correlate with Hepatocyte Magnesium Depletion
Source: Nutrients. 2023 Jun 3;15(11):2626. doi: 10.3390/nu15112626 (PMC10255194; doi:10.3390/nu15112626)

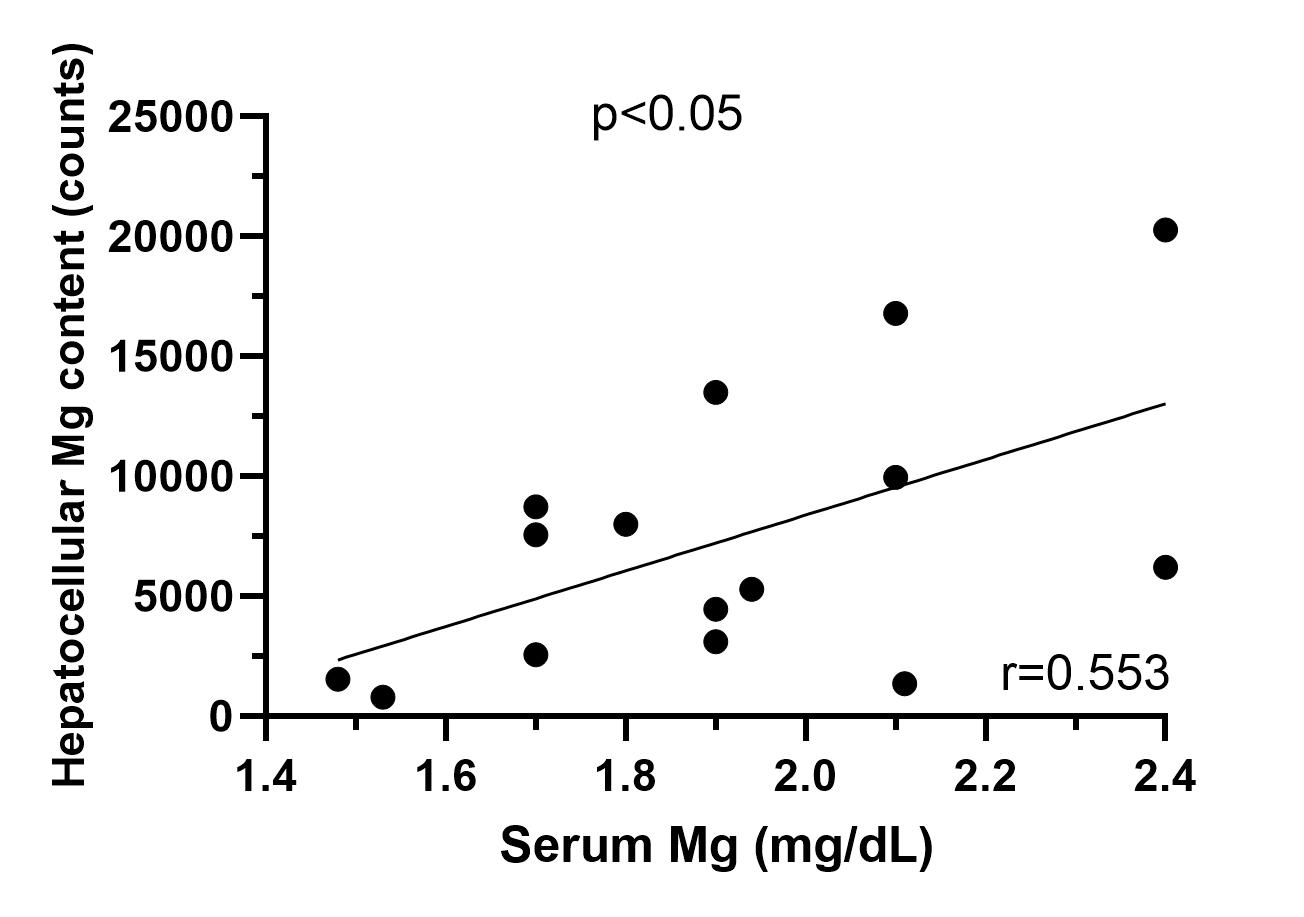

Supplement: Supplementary file 1 [file nutrients-15-02626-s001.zip › supplementary figure S1.jpg]
